# Antiviral Activity of Eugenol Against Chinese Rice-Field Eel Rhabdovirus in *Monopterus albus*

**DOI:** 10.3390/ani16020315

**Published:** 2026-01-20

**Authors:** Jingwen Jiang, Mingyang Xue, Wenzhi Liu, Yong Zhou, Yiqun Li, Yuding Fan

**Affiliations:** 1 Yangtze River Fisheries Research Institute, Chinese Academy of Fishery Sciences, Wuhan 430223, China; j178381682@outlook.com (J.J.); xmy@yfi.ac.cn (M.X.); liuwenzhialisa@yfi.ac.cn (W.L.); zhouy@yfi.ac.cn (Y.Z.); 2 College of Fisheries and Life Science, Shanghai Ocean University, Shanghai 201306, China

**Keywords:** eugenol, rhabdovirus, *Monopterus albus*, antiviral, apoptosis

## Abstract

Chinese rice-field eel rhabdovirus (CrERV) is a serious epidemic pathogen of Chinese rice-field eel and causes severe economic losses to aquaculture. However, there are no commercial drugs presently available to control CrERV infection. Eugenol is a bioactive compound extracted from clove plants and exhibits potential antiviral activity. In the study, the antiviral activity of eugenol against CrERV was investigated in Chinese rice-field eel (*Monopterus albus*). Eugenol reached the highest inhibition rate of 96.6% at 40 mg/L in Chinese rice-field eel kidney cells (CrEK). Notably, eugenol exhibits antiviral activity by directly targeting CrERV and additionally confers prophylactic effects against infection via its action on CrEK cells. The results of exploring the viral invasion cycle demonstrated that eugenol primarily exerted its antiviral effect during the middle stage and late stage (12 h and 24 h) of viral infection. In addition, eugenol inhibited CrERV-induced apoptosis of CrEK cells, maintained mitochondrial membrane potential levels, maintained physiological cellular morphology and structure, and protected cells from loss of cellular morphology, formation of apoptotic vesicles, and cell fragmentation. For the in vivo study, eugenol increased the survival rate of CrERV-infected rice-field eel by 56% and 48%, in prevention experiments and treatment experiments, respectively. Concurrently, eugenol significantly reduced viral loads and induced the upregulation of anti-inflammatory and antioxidant genes, indicating its potential for immunoregulation. In summary, eugenol holds potential for both preventing and treating CrERV infections in the aquaculture context.

## Simple Summary

Chinese rice-field eel rhabdovirus (CrERV) is a serious epidemic pathogen of Chinese rice-field eel and causes severe economic losses to aquaculture. Therefore, it is important to find an effective antiviral strategy to combat highly lethal CrERV outbreaks. Herbal plants have been widely used and play a vital role in disease treatment. This study aimed to evaluate the effect of the antiviral activity of eugenol against CrERV infection. The results demonstrated that eugenol can effectively inhibit CrERV replication both in vitro and in vivo. Furthermore, eugenol maintained cell morphology, attenuated CrERV-induced nuclear damage, and inhibited apoptosis by protecting mitochondrial membrane potential. It also improved the survival of Chinese rice-field eel, reduced viral load, and influenced CrERV infection by promoting the expression of antiviral-related genes. All the above results show that eugenol is a promising agent against CrERV in the fish industry.

## 1. Introduction

The Chinese rice-field eel, *Monopterus albus* (family, Synbranchidae; order, Synbranchiformes), is an important freshwater aquaculture species in China [1], highly valued by consumers for its fast growth rate, delicious taste, and high nutritional value [2]. However, as the scale of Chinese rice-field eel artificial breeding continues to expand, various disease problems have become increasingly prominent, causing serious economic losses to Chinese rice-field farming [3,4]. Chinese rice-field eel rhabdovirus (CrERV) was isolated and identified by Liu Wenzhi et al. in 2019 from diseased Chinese rice-field eels in Hubei Province, China [5]. The viral disease caused by CrERV can lead to more than 80% mortality, which has a serious impact on the production and economic benefits of the eel farming industry [5]. So far, no effective drugs, vaccines, or methods to control this disease have been discovered [6]. Therefore, it is imperative to develop an effective antiviral strategy to combat highly lethal CrERV outbreaks.

In ancient times, herbal plants were widely used and played a vital role in disease treatment. The majority of antiviral herbs were found to contain active components such as flavones, alkaloids, and polyphenols, which play an important role against viruses [7]. For instance, Saikosaponin D exhibits significant antiviral activity against spring viraemia of carp virus (SVCV) [8]. Artemisinin significantly inhibited SVCV replication, increased the survival rate of common carp by 33.3%, and markedly reduced viral load [9]. Esculin impedes Largemouth bass ranavirus (LMBRaV) infection by modulating viral entry mechanisms and enhances the survival rate of largemouth bass infected with LMBRaV, demonstrating potential for both prophylactic and therapeutic applications against LMBRaV infection in aquaculture settings [10]. In addition, ursolic acid (UA) showed strong anti-infectious hematopoietic necrosis virus (IHNV) activity in vivo, as indicated by increasing the survival rate of rainbow trout and inhibiting viral gene expression [11].

Eugenol is a phenolic aromatic compound obtained mainly from clove oil. Due to its known antibacterial, antiviral, antifungal, anticancer, anti-inflammatory, and antioxidant properties, it has long been used in various areas, such as medicine and pharmacology [12]. Studies have revealed that eugenol exhibits potential efficacy in the prevention and treatment of bacterial diseases in aquatic products [13]. Eugenol also exhibits potent anti-inflammatory and antioxidant properties. Eugenol is believed to regulate the cellular inflammatory cascades, including the NF-κB and ERK/MAPK pathways, and the release of proinflammatory interleukins [14]. In other studies, LPS (lipopolysaccharide)-induced lung inflammation was relieved by eugenol through a reduction in TNF-α and inhibition of NF-κB signaling, while also improving the alveolar damage [15]. Eugenol is capable of completely inhibiting iron ion- and Fenton reagent-induced lipid peroxidation [16]. In addition, eugenol exhibits significant antiviral activity. Studies have demonstrated that eugenol significantly inhibits herpes simplex-1 (HSV-1) and HSV-2 [17]. Eugenol also demonstrated antiviral activity against the influenza A virus (IAV) by inhibiting its replication [18]. Finally, it was also identified as an inhibitor of the Ebola virus in vitro [19]. In contrast, little is known about its antiviral activity against CrERV.

In this study, Chinese rice-field eel kidney (CrEK) cells were employed as an in vitro infection model for CrERV to evaluate the anti-CrERV activity of eugenol. Comprehensive analyses were conducted, including observation of cellular morphology, assessment of nuclear damage, measurement of mitochondrial membrane potential (MMP) alterations, and examination of ultrastructural integrity via scanning electron microscopy (SEM) and transmission electron microscopy (TEM). The antiviral efficacy of eugenol was further validated in vivo through RT-qPCR-based viral load quantification and survival assays in Chinese rice-field eel. Our findings demonstrate that eugenol exhibits potent inhibitory effects against CrERV infection, highlighting its feasibility as a promising candidate for antiviral therapeutics in aquaculture practices targeting CrERV-associated pathologies.

## 2. Materials and Methods

### 2.1. Cells, Virus, and Fish

The Chinese rice-field eel kidney (CrEK) cell line originated from the Department of Fish Diseases, Yangtze River Fisheries Research Institute, Chinese Academy of Fishery Sciences. Cells were maintained at 28 °C in Medium 199 (Hyclone, Logan, UT, USA) supplemented with 10% fetal bovine serum (FBS) (Yeasen, Shanghai, China). The CrERV virus used in this study was originally isolated in 2017 and is routinely propagated in CrEK cells.

Experimental Chinese rice-field eels (mean body weight: 4.2 ± 0.5 g) were obtained from a farm in Xiantao, Hubei Province. The rice-field eels were acclimated for 14 days in a 300 L recirculating system under the following conditions: water temperature at 25 ± 1 °C, twice-daily feeding, and a daily water exchange of approximately 30%. PCR analysis was used to confirm their CrERV-free status using CrERV-G primers (Table 1). Eugenol was purchased from Aladdin Chemistry (Shanghai, China, 97-53-0.CAS number). All animal experiments were approved by the Laboratory Animal Centre of the Yangtze River Fisheries Research Institute, Chinese Academy of Fishery Sciences (Ethical Approval ID: YFI2024-fanyuding-2205).

### 2.2. Eugenol Cytotoxicity Assay CCK-8

Assessment of eugenol’s impact on CrEK cell viability was conducted via the Cell Counting Kit-8 (Beyotime, Shanghai, China). Briefly, CrEK cells were seeded into 96-well plates (Labselect, Beijing, China) at a density of 1 × 10^4^ cells per well and allowed to adhere for 24 h. Subsequently, the cells were treated with a series of eugenol dilutions. Following a 48 h treatment period, cytotoxicity was quantified according to the manufacturer’s instructions for the CCK-8 assay. Measurement of absorbance at 450 nm was performed using a microplate reader (Biotek, Winooski, VT, USA).

### 2.3. Eugenol for CrERV Duplication and CPE Reduction Assay

Eugenol was prepared at six different concentrations in cell maintenance medium. Dimethyl sulfoxide (DMSO) (0.10%, *v*/*v*) served as the positive control. Following this preparation, cells were inoculated with CrERV at a dose of 10^3^ × TCID_50_/mL and incubated at 28 °C for 2 h. Subsequently, the inoculum was replaced with the eugenol-containing maintenance medium. After 48 h of culture, cells were harvested to perform qRT-PCR. This analysis aimed to quantify the expression of the CrERV glycoprotein (G) gene and thereby evaluate the compound’s antiviral efficacy.

### 2.4. Co-Treatment and Pretreatment Assay

A mixture containing CrERV (10^3^ TCID_50_) and eugenol (40 mg/L) was prepared and incubated at 28 °C for 0, 15, 30, and 60 min. This mixture was then applied to CrEK monolayers in 12-well plates. Following a 2 h adsorption period, the inoculum was replaced with fresh cell maintenance medium. Cells were harvested 48 h post-infection for subsequent qRT-PCR quantification of viral glycoprotein gene expression.

CrEK cells were pretreated with eugenol for 8, 16, or 24 h. Subsequently, the cells were washed twice with sterile PBS and challenged with CrERV (10^3^ × TCID_50_/mL) for 2 h. After a total infection period of 48 h, the expression of the CrERV-G gene was quantified by RT-qPCR. In both assays, 0.1% (*v*/*v*) DMSO was used as the vehicle control at all time points.

### 2.5. Time-of-Addition Assay

For the time-of-addition assay, we followed the same protocol outlined above [23]. Following a 2 h adsorption period, the CrERV inoculum was removed and replaced with cell maintenance medium. Eugenol (40 mg/L) was then applied to the infected cells for a 2 h pulse at various designated time points across the viral replication cycle. Regardless of treatment timing, all cells were harvested at 24 h post-infection (hpi) for subsequent qRT-PCR analysis.

### 2.6. Cell Nuclear Damage Assay

For nuclear damage assessment, we first plated CrEK cells in confocal dishes and cultured them for 24 h. The cells were subsequently infected with CrERV at 1 × 10^3^ TCID_50_/mL for 2 h, after which the medium was changed to one supplemented with 40 mg/L eugenol. Following a 48 h treatment, cells were washed three times with 0.1 M PBS, stained with 2-(4-Amidinophenyl)-6-indolecarbamidine dihydrochloride (DAPI) (1 mg/L) and 1,1′-dioctadecyl-3,3,3′,3′-tetramethylindocarbocyanine perchlorate (Dil) (5 mg/mL), and finally visualized under a Leica DM5000 confocal microscope (Wetzlar, Germany).

### 2.7. Detection of Mitochondrial Membrane Potential

CrEK cells were seeded into confocal dishes and allowed to adhere for 24 h. Subsequently, the cells were infected with CrERV at a concentration of 10^3^ TCID_50_/mL for 2 h. Following infection, fresh culture medium containing 40 mg/L eugenol was added to the cells. After 48 h of incubation, the cells were stained with JC-1 to assess mitochondrial membrane potential. Fluorescence was observed using a confocal microscope (Olympus FV 3000, Tokyo, Japan).

### 2.8. Ultrastructural Observation

For ultrastructural analysis, cells were cultured on sterile coverslips in 6-well plates for 24 h prior to further processing. Subsequently, they were infected with CrERV (1 × 10^3^ TCID_50_/mL) for 2 h. The inoculum was then replaced with fresh maintenance medium containing 40 mg/L eugenol. Following a 48 h infection period, cells were collected and subjected to overnight fixation in 2.5% glutaraldehyde at 4 °C. For ultrastructural examination, samples were prepared for and viewed under a Hitachi SU8010 scanning electron microscope (Tokyo, Japan). In parallel, ultrathin sections were cut and imaged with a Hitachi H7650 transmission electron microscope (Tokyo, Japan), in accordance with established methodologies [24,25].

### 2.9. Anti-CrERV Activities of Eugenol In Vivo

#### 2.9.1. Toxicity Assessment of Eugenol to Chinese Rice-Field Eels

To evaluate the in vivo toxicity of eugenol in rice-field eels, experimental feeds were prepared by incorporating eugenol at doses of 20, 40, and 60 mg/kg. A control group received feed mixed with an equivalent volume of DMSO. The study employed a completely randomized design with three replicate tanks per treatment, each containing 30 rice-field eels. Mortality was recorded daily over a 14-day period. Additionally, feeding behavior and swimming activity were monitored throughout the experiment. At the endpoint (day 14), surviving rice-field eels were dissected for gross pathological examination of their internal organs.

#### 2.9.2. The Protective Effects of Eugenol on CrERV-Infected Chinese Rice-Field Eels

A protective efficacy trial was conducted in CrERV-infected rice-field eels. Fish were randomly allocated to three treatment arms: an uninfected control (M199 injection), an infected control (CrERV injection + DMSO diet), and a treatment group (CrERV injection + eugenol diet at 40 mg/kg). Each group had three replicates of 30 fish. All injections (0.1 mL of virus suspension at 10^7^ × TCID_50_/mL or M199) were administered intraperitoneally, followed by the respective dietary treatments 12 h later. Mortality was tracked daily for 14 days. In parallel, a viral kinetics study was performed. For this purpose, infected fish under DMSO or eugenol diet were sampled at 4, 7, and 10 dpi (*n* = 3 pools per group per time point) to quantify tissue viral load by qRT-PCR.

#### 2.9.3. Preventative Effects of Eugenol on CrERV Infection in Chinese Rice-Field Eels

A prevention study was conducted to assess whether eugenol pretreatment could protect against CrERV infection. Fish were assigned to three groups: Control, CrERV, and eugenol +CrERV. Prior to viral challenge, the eugenol +CrERV group was fed a diet containing 40 mg/kg eugenol for 14 days, whereas the other two groups received a DMSO-control diet. Subsequently, the CrERV and eugenol +CrERV groups were intraperitoneally injected with CrERV (10^7^ × TCID_50_/mL in 0.1 mL), and the control group received 0.1 mL M199. Survival rates were recorded, and tissues were harvested at 4, 7, and 10 dpi for analysis of viral replication and host antiviral responses.

### 2.10. Total RNA Extraction and Gene Quantification

Total RNA was isolated using a dedicated extraction kit (Yeasen, Shanghai, China). cDNA was then synthesized according to the protocol of the Hifair^®^ III 1st Strand cDNA Synthesis SuperMix for qPCR (Yeasen, Shanghai, China) and stored at −20 °C until analysis. qRT-PCR was carried out on a Rotor-Gene Q instrument (QIAGEN, Dusseldorf, Germany) with Hieff UNICON^®^ Universal Blue qPCR SYBR Green Master Mix (Yeasen, Shanghai, China). The primer sequences used are provided in Table 1. PCR was performed with the following profile: 95 °C for 5 min, and then 40 cycles of 95 °C for 10 s, 60 °C for 20 s, and 72 °C for 20 s. Gene expression data were analyzed via the 2^−ΔΔCt^ method.

### 2.11. Statistical Analysis

Data analysis and visualization were conducted using GraphPad Prism 9 (GraphPad Software, San Diego, CA, USA). Data are expressed as mean ± standard deviation (SD). Statistical significance was defined as *p* < 0.05. All experiments were performed with at least three independent biological replicates.

## 3. Results

### 3.1. In Vitro Antiviral Activity of Eugenol

**Figure 1:**
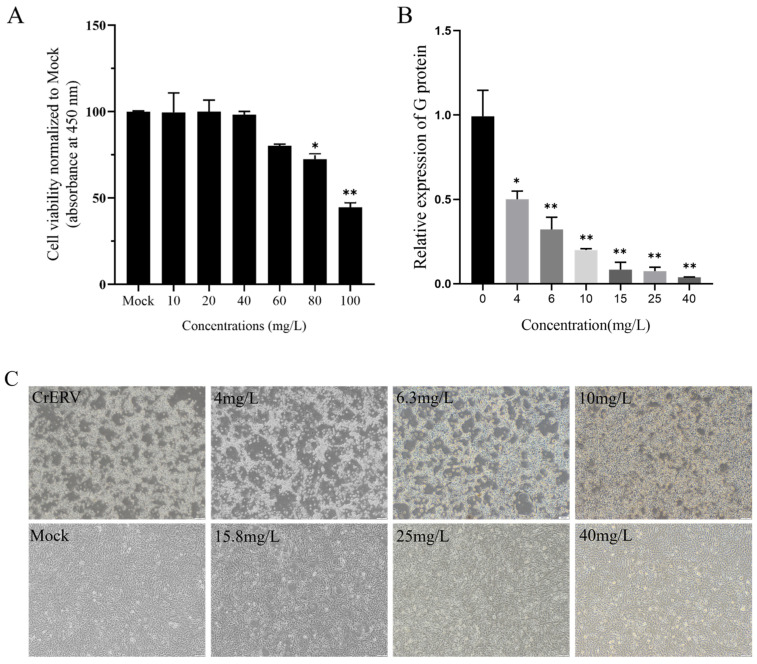
Inhibitory effects of eugenol on CrERV in vitro. (**A**) The cytotoxic effects of eugenol on CrEK cells. Each value was presented as mean ± SD, normalized to untreated values. (**B**) Six-point dose–response curves reflecting the antiviral activity of eugenol in CrEK cells. qRT-PCR analysis of G protein expression. (**C**) Morphological effects of eugenol on CrERV infection in CrEK cells. All images were captured at the same magnification. ** *p* < 0.01, * *p* < 0.05.

We tested the toxicity of the eugenol on CrEK cells and evaluated its anti-CrERV activity. We assessed the toxicity of eugenol on CrEK cells using the CCK-8 method. According to cytotoxicity tests, 40 mg/L was selected as the safe concentration of the drug for subsequent experiments (Figure 1A). Subsequently, qRT-PCR was employed to analyze the impact of eugenol on CrERV-G gene expression across six concentrations. The results exhibited a dose-dependent inhibition of eugenol on CrERV and eugenol with a maximum anti-CrERV rate of 96.6% at the concentration of 40 mg/L (Figure 1B). The cytopathic effect (CPE) of CrERV-infected cells was strongly reduced after 48 h in the presence of eugenol (Figure 1C).

### 3.2. Effects of Eugenol on CrERV Infectivity

**Figure 2:**
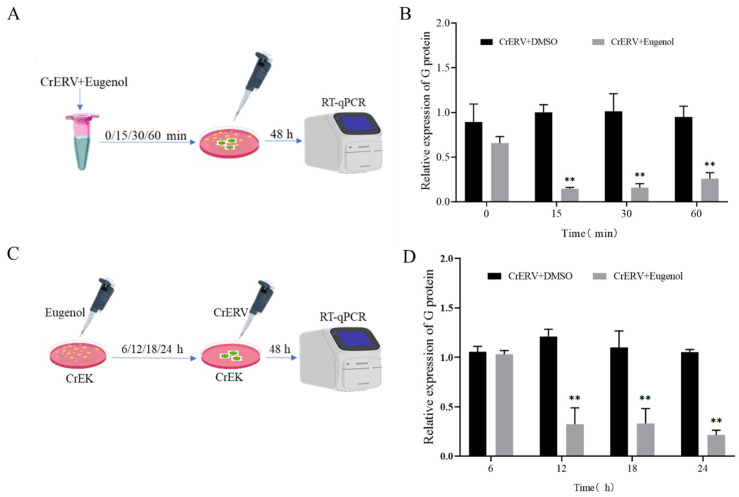

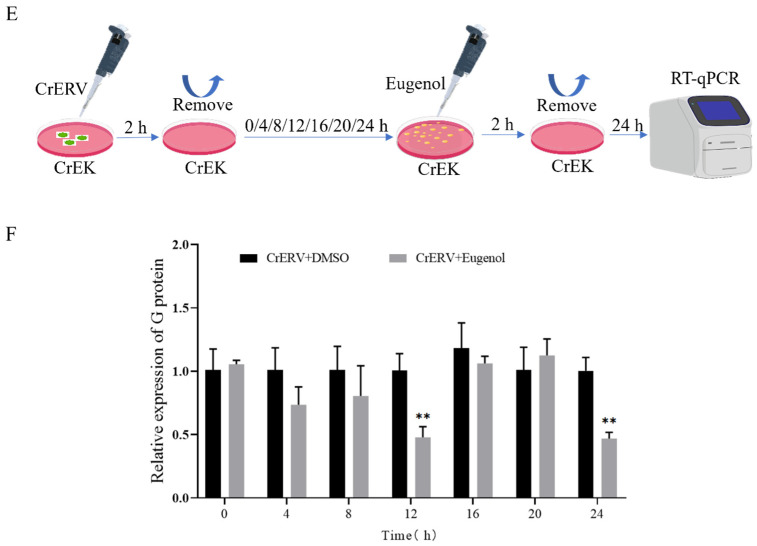
The effect of eugenol on CrERV infection was assessed. (**A**) Workflow schematic of CrERV infectivity assay. (**B**) Alterations in CrERV-G protein gene expression following 15, 30, and 60 min of viral incubation with eugenol, and 48 h of infection in CrEK cells. (**C**) Workflow schematic for cell pretreatment tests. (**D**) Changes in CrERV-G gene expression after 6, 12, 18, and 24 h of eugenol pretreatment. (**E**) Time-of-addition assay workflow schematic. (**F**) Relative G gene RNA levels were determined by RT-qPCR from the addition assay. Experiments were performed in triplicate, and each value represented the mean ± SD. ** *p* < 0.01.

We first investigated the possibility that eugenol could really destroy viral particles, as shown in Figure 2A. CrERV was pre-incubated with eugenol at 28 °C for durations of 0, 15, 30, and 60 min prior to infection. After 15, 30, and 60 min of incubation, the gene expression level of CrERV-G protein in the eugenol group was only 0.14-fold, 0.16-fold, and 0.27-fold (*p* < 0.01) compared with that in the DMSO-treated group (Figure 2B). Regarding whether eugenol can be used to prevent CrERV, as demonstrated in Figure 2C, as expected, eugenol enhanced the antiviral response at all three incubation time points. After 12 and 18 h of eugenol treatment, the viral load decreased by 73.5% and 76.9%, respectively, while the viral inhibition rate reached 83.7% after 24 h (*p* < 0.01, Figure 2D). To further investigate the mechanism of eugenol against CrERV, the time-of-addition assay was tested (Figure 2E). The results showed that the addition of eugenol at 12 h and 24 h post-viral infection significantly inhibited CrERV replication. At 12 h, the expression level of the G protein decreased by 52.7%, and the viral inhibition rate was 47.6% (*p* < 0.01). At 24 h, the expression level of the G protein decreased by 53.5%, and the viral inhibition rate was 46.7% (*p* < 0.01, Figure 2F).

### 3.3. Nucleus Damage Reduction Effect of Egenol

**Figure 3:**
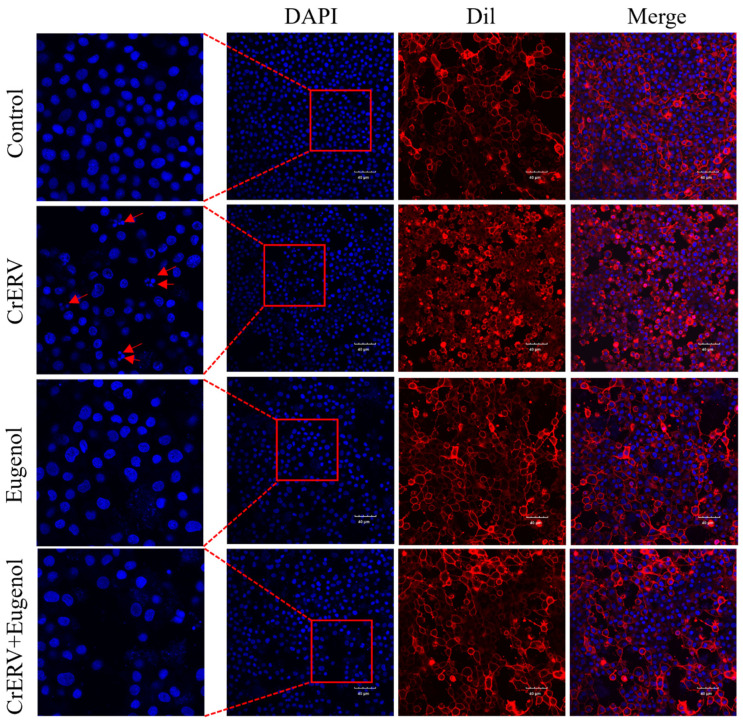
Eugenol reduced the quantity of apoptosis body in CrEK cells. Blue color represents the nucleus, red represents the cell membranes, and red arrows indicate the apoptotic body.

Fluorescence observation of Dil/DAPI-stained CrEK cells validated that eugenol exerted an anti-apoptotic effect. As depicted in Figure 3, CrERV-infected cells displayed typical apoptotic traits, including altered cell morphology and nuclear fragmentation. On the other hand, eugenol-treated cells exhibited diminished apoptotic characteristics, a decrease in the proportion of apoptotic vesicles, and preserved nuclei with a normal spindle shape. Therefore, eugenol attenuated CrERV-induced apoptosis, further confirming its inhibitory effect on CrERV.

### 3.4. Eugenol Protects Mitochondrial Functions of CrEK Cells During CrERV Treatment

**Figure 4:**
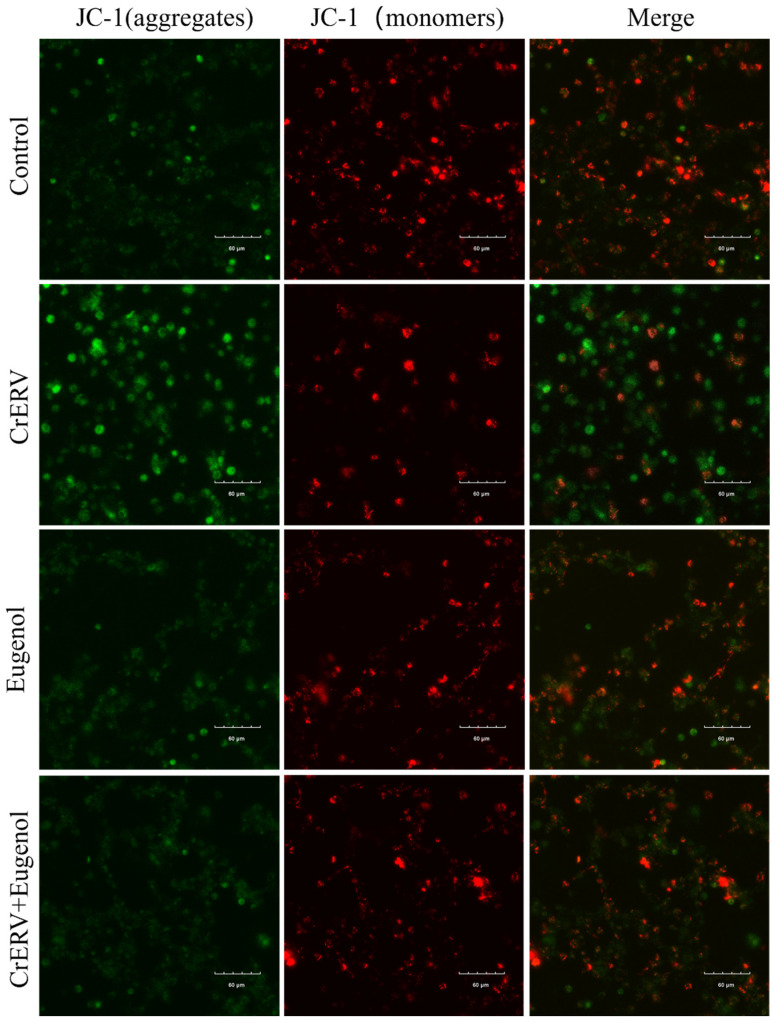
Effects of eugenol on CrERV-induced mitochondrial membrane potential. CrEK cells are treated in the presence or absence of 40 mg/L eugenol with CrERV infection at 48 h.

JC-1 is an ideal fluorescent probe that is widely used for detecting mitochondrial membrane potential. Under high membrane potential conditions, it forms aggregates that emit red fluorescence, while under low membrane potential conditions, it exists as monomers that produce green fluorescence [26]. The results showed that CrERV induced a significant decrease in mitochondrial membrane potential (Figure 4). However, in CrERV-infected cells treated with eugenol, the mitochondrial membrane potential was restored to levels comparable to the control group, and the virus-induced adverse effects were significantly suppressed. These results suggest that eugenol could inhibit virus-induced apoptosis by protecting mitochondrial membrane potential, thus playing an antiviral role.

### 3.5. Eugenol Had a Morphologically Protective Effect on CrEK Cells

**Figure 5:**
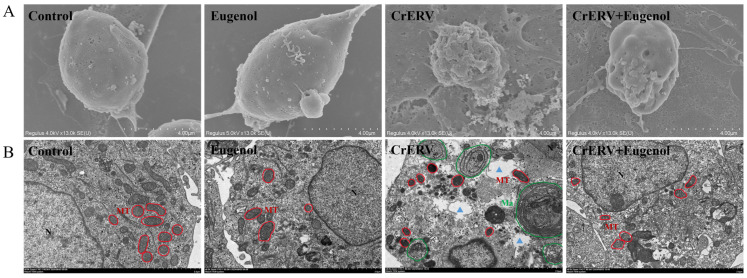
Morphological and ultrastructural analyses of eugenol in cells infected with CrERV. (**A**) Scanning electron microscopy image showing morphological effects of CrERV infection and antiviral activity of 40 mg/L eugenol at 48 h. (**B**) Ultrastructural modifications in CrERV particles and CrEK cells after 48 h of treatment with 40 mg/L eugenol. Mitochondria are highlighted in red and autophagosomes are highlighted in green, while cytoplasmic vacuolization is marked with blue triangles.

In scanning electron microscopy (SEM) images of CrERV-infected cells (Figure 5A), typical apoptotic features were observed, including cell surface rupture and loss of cellular morphology. In contrast, cells in the control group and the eugenol-treated group exhibited normal and intact morphology with relatively smooth surfaces. Meanwhile, treatment with eugenol at 48 h post-CrERV infection significantly reduced the damage caused by CrERV to CrEK cells. Furthermore, transmission electron microscopy (TEM) images revealed that (Figure 5B) CrERV-infected cells exhibited typical apoptotic features, including chromatin condensation, mitochondrial shrinkage, disappearance of cristae (highlighted in red in Figure 5B), cytoplasmic vacuolization (blue triangles in Figure 5B), and a significant increase in autophagosomes (highlighted in green in Figure 5B). In contrast, treatment of CrERV-infected cells with eugenol markedly reduced the number of autophagosomes and protected the nucleus, mitochondria, and other organelles compared to the virus-infected group. These findings demonstrate that eugenol can effectively inhibit CrERV-induced apoptotic characteristics in CrEK cells.

### 3.6. Protective Effects of Eugenol on CrERV-Infected Rice-Field Eels

Prior to the fish experiment, we evaluated the toxicity of eugenol to rice-field eels by using clinical symptoms. No symptoms of rice-field eels were observed when the concentration of eugenol was 40 mg/kg, and the Chinese rice-field eels were normal in eating and swimming, and no abnormalities in viscera were found in their anatomy. In contrast, a concentration of 60 mg/kg exhibited notable toxicity effects in Chinese rice-field eels, characterized by diminished food consumption.

**Figure 6:**
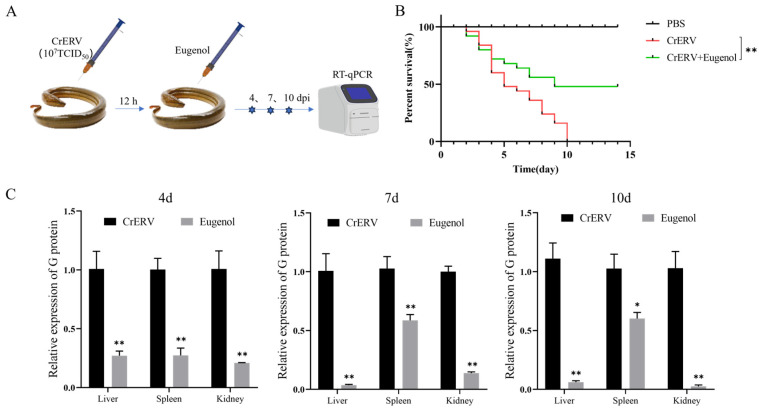
Effects of eugenol on survival rate and viral loads in CrERV-infected rice-field eels. (**A**) Workflow schematic for survival and viral load analysis. (**B**) Daily monitoring of mortality in CrERV-infected rice-field eels. (**C**) Viral load of rice-field eels’ liver, spleen, and kidney. Each value is the mean ± SD. * *p* < 0.05, ** *p* < 0.01.

The mortality rate of the virus-treated group was as high as 100% within 14 days after infection, and the survival rate of CrERV-infected rice-field eels increased to 48% (*p* < 0.01) after treatment with eugenol (Figure 6B). Furthermore, an examination of the viral content in the liver/spleen/kidney tissue was conducted on days 4, 7, and 10 post-infection. RT-qPCR results showed that the viral content in the liver, spleen, and kidney tissue of rice-field eels was reduced, and treatment exhibited a noteworthy reduction compared to the CrERV_DMSO_ group across all time points (Figure 6C). Consequently, these findings affirm the therapeutic potential of eugenol against CrERV infection in Chinese rice-field eels.

### 3.7. Preventative Effects of Eugenol on CrERV Infection

**Figure 7:**
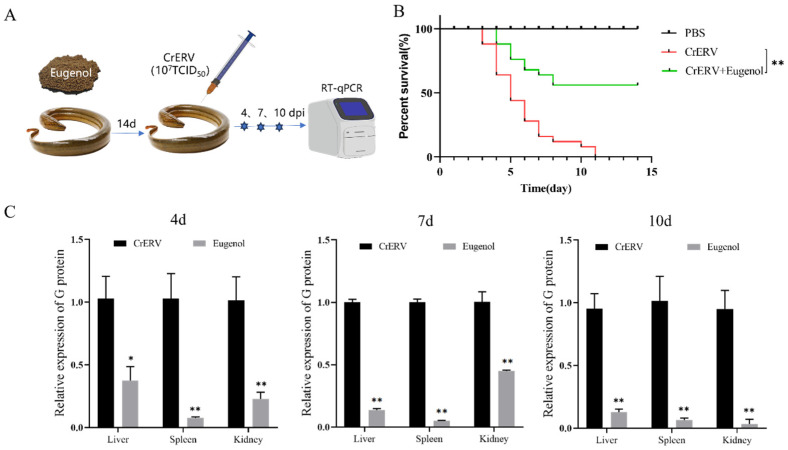
Effects of eugenol pretreatment on rice-field eels. (**A**) Workflow schematic for survival and viral load analysis. (**B**) Survival rate of rice-field eels infected with CrERV. (**C**) Viral load of rice-field eels in liver, spleen, and kidney. Each value is the mean ± SD. * *p* < 0.05, ** *p* < 0.01.

After observing the favorable effects of eugenol treatment on CrERV-infected rice-field eels, we proceeded to examine the inhibitory effects of eugenol pretreatment against viral infection in vivo. Throughout the observation period, there was a notable difference (*p* < 0.01) in the survival rate between the eugenol treatment group and the CrERV-infected group. Notably, eugenol pretreatment increased the survival rate by 58% following the rice-field eels infected with CrERV. Additionally, the viral load in the liver, spleen, and kidney exhibited significant reduction on days 4, 7, and 10 after infection (*p* < 0.01, Figure 7).

### 3.8. Effects of Eugenol on Expression of Antiviral-Related Genes

**Figure 8:**
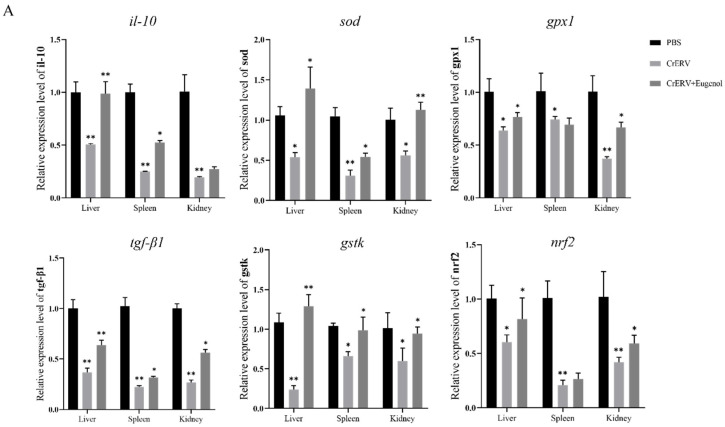

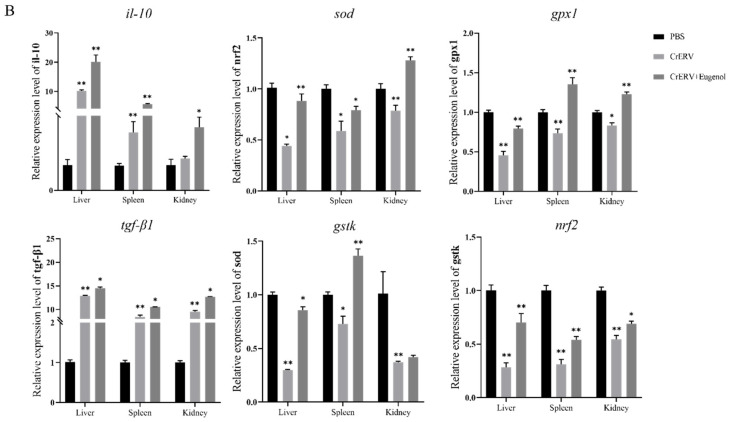
Eugenol alters gene expression associated with antiviral resistance in the liver, spleen, and kidney of rice-field eels. Anti-inflammatory and antioxidant genes, including *il-10*, *tgf-β1*, *sod*, *gpx1*, *gstk*, and *nrf2*, were determined by RT-qPCR at 7 d post-infection. (A) In the pre-exposure eugenol group, gene expression changes were observed in the rice-field eels. (B) In the post-exposure eugenol group, gene expression changes were also detected in the rice-field eels. Data are expressed as mean ± SD. ** *p* < 0.01, * *p* < 0.05.

To determine whether the antiviral effects of eugenol on rice-field eels are associated with their anti-inflammatory and antioxidant activities, we examined the expression of genes related to anti-inflammatory and antioxidant activities on day 7 post-CrERV infection. The results showed that, compared to the PBS group, the expression levels of anti-inflammatory genes (*il-10*, *tgf-β1*) and antioxidant genes (*sod*, *gpx1*, *gstk*, and *nrf2*) were significantly downregulated in CrERV-infected rice-field eels, except for the upregulation of *il-10* and *tgf-β1* in the post-exposure eugenol group. However, in both the pre-exposure eugenol groups and post-exposure eugenol groups, the expression levels of these genes were upregulated in CrERV–eugenol-treated rice-field eels (Figure 8). In the pre-exposure eugenol group, the upregulation of anti-inflammatory and antioxidant gene expression was most pronounced in the liver compared to the CrERV group, with *il-10*, *sod*, and *gstk* expression levels increasing by 1.96-fold, 2.62-fold, and 5.33-fold, respectively (Figure 8A). A similar trend was observed in the spleen and kidney. In the post-exposure eugenol group, compared to the CrERV group, the expression levels of *il-10* in the liver, spleen, and kidney were upregulated by 1.97-fold, 2.39-fold, and 2.01-fold, respectively (Figure 8B), while *sod* expression levels increased by 2.93-fold, 1.88-fold, and 1.13-fold, respectively. In conclusion, CrERV infection led to the downregulation of anti-inflammatory and antioxidant genes in rice-field eels, whereas eugenol alleviated this effect by significantly upregulating the expression levels of these genes.

## 4. Discussion

As an important freshwater aquaculture species in China, the industrial scale of the Chinese rice-field eel is huge, and the annual output value has exceeded 10 billion yuan [27]. Virus outbreaks can lead to mass deaths, and farmers could face significant losses. CrERV is a virus that has been gaining traction in aquaculture in recent years and poses a significant threat to the Chinese rice-field eel farming industry in particular. At present, the prevention and control of CrERV relies on routine measures, such as disinfection and isolation. Eugenol, as a natural phenolic compound, is widely found in plant essential oils, and its insecticidal, antibacterial, anti-inflammatory, antiviral, wound healing, antioxidant, and anticancer properties have all been described. At present, eugenol is mainly used as an anesthetic in aquaculture, and there are few studies on it as an antiviral. As a natural antiviral, eugenol has great potential in the development of antiviral drugs. Therefore, we conducted a study on the antiviral activity of eugenol against CrERV.

We first determined the cytotoxicity of eugenol on CrEK cells, establishing its maximum safe concentration at 40 mg/L. Previous studies have shown that the maximum safe concentration of arctigenin in crucian Gibel carp brain (GiCB) is 2 mg/L [6], while that of artemisinin in Epithelioma papulosum cyprini (EPC) cells is 100 μM [9]. Unlike many natural compounds that may exhibit cytotoxicity at higher concentrations, eugenol demonstrates low toxicity to host cells within its effective antiviral concentration range. Epigallocatechin-3-gallate (EGCG) extracted from Green Tea disrupted the structure of viral particles and also affected the binding of viral particles to cell receptors, as well as viral invasion into host cells [28]. Arctigenin has a preventive effect against Micropterus salmoides rhabdovirus (MSRV) [29]. Our concentration gradient experiments revealed that, in vitro, eugenol significantly inhibits CrERV expression, exhibiting an antiviral response exceeding 96.6% at 40 mg/L. Furthermore, our research found that eugenol directly interferes with the interaction between CrERV and CrEK cells, reducing the virus’s ability to infect host cells. Pretreatment experiments demonstrated that eugenol has a prophylactic effect against CrERV infection.

Viruses initiate their life cycle by attaching to host cell surface receptors, entering cells, uncoating viral nucleic acids, and replicating genomes [30]. Liu et al. evaluated the anti-CrERV activity of arctigenin and demonstrated that it primarily inhibits the early stages of CrERV replication and viral particle adsorption [6]. Saikosaponin C primarily exerted its antiviral effect during the middle stage (2–6 h) of LMBRaV viral infection [31]. In our experiment involving eugenol pre-incubation followed by CrERV infection, we observed that a 6 h pre-incubation was ineffective, while longer pre-incubation periods (12/18/24 h) were effective. We speculate that eugenol may pre-activate the innate antiviral state of host cells, suggesting that a sufficient “preparation” time is required for eugenol to exert optimal protective effects. Furthermore, eugenol effectively suppressed CrERV proliferation at 12 h post-infection, with its inhibitory effect diminishing thereafter before resuming at 24 h. We deduce that eugenol targets not only viral entry but also subsequent replication and assembly processes. Specifically, the strong suppression observed at 12 h may correspond to the viral genome integration phase, while the secondary inhibition at 24 h could be associated with virion assembly. This time-dependent antiviral pattern shares similarities with flavonoid-mediated HIV inhibition. Richman [32] reported that flavonoids, in addition to targeting reverse transcriptase, also modulate multiple steps of the HIV-1 life cycle, including entry, integration, and maturation, a phenomenon analogous to the biphasic inhibition observed with eugenol in this study. These findings further support the potential of eugenol as a broad-spectrum antiviral agent.

Apoptosis is a programmed cell death process typically triggered by endogenous or exogenous signals, involving a series of complex molecular mechanisms [33]. Mitochondria play a central role in regulating apoptosis, with the reduction in mitochondrial membrane potential serving as a critical hallmark in the early stages of apoptotic cell death [34]. Studies have shown that dictamnine effectively inhibits NNV-induced CPE and reduces viral titers in vitro while preventing mitochondrial dysfunction through maintaining membrane potential [35]. Upon SVCV infection, EPC cells exhibited severe shrinkage with typical apoptotic characteristics, whereas cells treated with coumarin compounds showed no significant alterations in their microscopic morphology. More importantly, the coumarin compounds were found to upregulate antioxidant enzyme gene expression, activate the Nrf-2 pathway, and maintain intracellular redox homeostasis [36]. Additionally, chlorogenic acid has been demonstrated to inhibit viral release by directly targeting apoptosis induced by PDCoV infection [37]. Similarly, we observed that CrEK cells infected with CrERV exhibited typical apoptotic characteristics, including nuclear fragmentation, formation of apoptotic bodies, and a significant decrease in mitochondrial membrane potential, all of which are highly consistent with classical features of apoptosis. Eugenol treatment inhibited CrERV-induced mitochondrial membrane potential depolarization and significantly suppressed nuclear damage.

Furthermore, ultrastructural analysis revealed that CrERV-infected CrEK cells displayed chromatin condensation, mitochondrial shrinkage, cytoplasmic vacuolization, and increased autophagosome formation, morphological alterations consistent with both apoptosis and autophagy. These findings indicate that CrERV infection not only induces apoptosis but also triggers autophagy in host cells. Autophagy is a cellular self-degradation process that eliminates damaged organelles and proteins, yet under certain conditions, it may also facilitate cell death [38]. Previous studies have demonstrated that eugenol effectively suppresses apoptosis by preserving mitochondrial function and downregulating apoptosis-related protein expression [39]. Furthermore, reactive oxygen species (ROS) and lipid peroxidation serve as critical triggers of apoptosis [40]. Research has shown that eugenol inhibits lipid peroxidation (with an IC_50_ of approximately 80 μM), including iron- and hydroxyl radical-induced lipid peroxidation in rat liver mitochondria [41]. Therefore, we believe that eugenol’s inhibition of CrERV-induced apoptosis is related to these mechanisms.

Some natural compounds have been proven to lead to increased survival and disease resistance in aquaculture. Intramuscular injection of genipin significantly inhibits WSSV infection, blocks viral replication, and increases the survival rate of WSSV-infected crayfish to 56.67% [42]. Artemisinin was found to significantly inhibit SVCV replication. Post-incubation administration by feeding increased the survival rate of common carp by 33.3% [9]. Given that the potent anti-CrERV activity of eugenol was demonstrated in vitro, we further investigated its inhibitory effects against CrERV infection in vivo, conducting preliminary studies to evaluate both prophylactic and therapeutic potential of eugenol in Chinese rice-field eels infected with CrERV. Our results showed that CrERV infection caused a high mortality rate (100%) in Chinese rice-field eels. Gratifyingly, eugenol was also highly effective in the treatment of CrERV infection in Chinese rice-field eels. The results demonstrated that eugenol exhibited a 56% prophylactic protection rate and a 48% therapeutic protection rate against CrERV infection. Concurrently, eugenol treatment significantly reduced viral load in infected Chinese rice-field eels.

Studies have demonstrated that artemisinin significantly suppresses SVCV replication by inducing the upregulation of antiviral-related genes [9]. Similarly, esculin enhances host immune responses through stimulating the expression of antiviral-associated genes [10], while coumarin derivatives inhibit white spot syndrome virus (WSSV) replication by increasing the expression of genes related to antimicrobial peptides [43]. Consistent with these findings, our study revealed that eugenol promotes the expression of both anti-inflammatory and antioxidant genes, suggesting its antiviral mechanism involves the upregulation of relevant gene expressions. Notably, eugenol exhibited superior efficacy as a prophylactic agent compared to its therapeutic application against CrERV infection. This finding indicates that eugenol can be used as a feed additive for preventive feeding in CrERV epidemic areas, or as a therapeutic agent in the early stages of infection.

## 5. Conclusions

In conclusion, the present study indicates that eugenol can effectively inhibit CrERV replication both in vitro and in vivo. Furthermore, eugenol maintained cell morphology, attenuated CrERV-induced nuclear damage, and inhibited apoptosis by protecting mitochondrial membrane potential. It also improved the survival of Chinese rice-field eel, reduced viral load, and influenced CrERV infection by promoting the expression of antiviral-related genes. All the above results show that eugenol is a promising agent against CrERV in the fish industry.

## Figures and Tables

**Table 1:** List of primers used in this study.

Gene	Primer Sequences (from 5′ to 3′)	Reference
CrERV-Fq	GCAAGCTTCCAAGGCCACTT	[20]
CrERV-Rq	GCAGACAGCACGTCCGATTC	
*Monopterus albus* EF1α-F	ATCCGTCGTGGATATGTGGC	[21]
*Monopterus albus* EF1α-R	AGCACTGGGGCATAACCTTC	
*il-10*-F	TTTGCCTGCCAAGTTATGAG	[22]
*il-10*-R	CATTTGGTGACATCGCTCTT	
*tgf-β1*-F	AACCCACTACCTCACTACCCG	[22]
*tgf-β1*-R	GCCGAAGTTGGAAACCCT	
*nrf2*-F	CTTCAGACAGCGGTGACAGG	[22]
*nrf2*-R	GCCTCATTCAGTTGGTGCTT	
*gstk*-F	TTGATGTTCCCCTGCGTTAT	[22]
*gstk*-R	CACCTGCTCTACCTGCTTGTC	
*gpx1*-F	GTTCACCGCCAAACTCTT	[22]
*gpx1*-R	TTCCCATTCACATCTACCTT	
*sod*-F	AGCTGGCTAAGTTCTCATTCAC	[22]
*sod*-R	GCAGTAACATTGCCCAAGTCT	

## Data Availability

The original contributions presented in this study are included in the article. Further inquiries can be directed to the corresponding authors.
